# Novel blood biomarkers for heart failure mortality: mechanistic pathways, aging biology, and risk stratification

**DOI:** 10.3389/fcvm.2026.1936855

**Published:** 2026-09-14

**Authors:** Xiaofeng Zeng, Wenwu Cheng, Yan Jiang, Li Mo

**Affiliations:** 1 Health Management Center of West China Hospital, Sichuan University, Chengdu, China; 2 Department of Integrated Therapy, Fudan University Shanghai Cancer Center, Shanghai, China; 3 Department of Oncology, Shanghai Medical College, Fudan University, Shanghai, China; 4 Evidence-Based Nursing Research Laboratory, West China Hospital, Sichuan University/West China School of Nursing, Sichuan University, Chengdu, China

**Keywords:** biomarkers, heart failure, mortality, pathophysiology, precision medicine, risk stratification

## Abstract

Heart failure (HF) is a major global health burden associated with high mortality after decompensation, particularly in older patients with multimorbidity. Although established biomarkers such as natriuretic peptides remain essential for initial risk stratification, they capture only a fraction of mortality risk in biologically heterogeneous aging populations. Because HF-related death arises from diverse pathophysiological mechanisms, no single biomarker can capture this mechanistic diversity. This narrative review synthesizes current evidence on circulating biomarkers associated with HF mortality and maps these molecules to specific pathways of cardiac aging and systemic disease. Aging fundamentally reshapes biomarker interpretation: chronic low-grade inflammation, increased vascular stiffness, impaired organ clearance, and endocrine remodeling can alter baseline biomarker concentrations and modify the clinical significance of their longitudinal changes. The evidence reviewed here provides a rationale for a mechanism-driven, pathway-based framework rather than the pursuit of a single prognostic predictor. This framework should be regarded as conceptual and hypothesis-generating. Key biomarker domains include fibro-inflammatory remodeling and myocardial stress, represented by soluble suppression of tumorigenicity 2, galectin-3, growth differentiation factor 15, and heart-type fatty acid-binding protein, which provide overlapping information on myocardial strain, fibrosis, and systemic stress. Markers of congestion, endothelial dysfunction, and vascular aging, including carbohydrate antigen 125, bioactive adrenomedullin, mid-regional pro-adrenomedullin, and endothelin-1, may provide complementary information on hemodynamic, serosal, right-sided, and vascular stress. Markers of cardiorenal and multiorgan reserve, including neutrophil gelatinase-associated lipocalin, albumin, and composite renal or nutritional indices, highlight that HF mortality may reflect systemic vulnerability rather than isolated ventricular dysfunction. Metabolic stress and inflammation, reflected by fibroblast growth factor 21, amino-acid metabolites, and inflammatory mediators, may help characterize metabolic exhaustion and frailty trajectories. Finally, proteomic and metabolomic approaches are promising tools for identifying molecular phenotypes, although assay heterogeneity, insufficient age-specific validation, uncertain incremental clinical utility, and limited bedside interpretability remain barriers. In conclusion, HF mortality may arise from the convergence of myocardial injury and systemic biological aging. Prospective biomarker-guided studies in older, multimorbid HF populations are required before pathway-based panels can be routinely used to inform treatment and care planning.

## 1 Introduction

Heart failure (HF) remains a major global health problem characterized by rising prevalence, a heavy hospitalization burden, and substantial early mortality following decompensation (1, 2). The contemporary HF population is growing older and increasingly multimorbid, with marked biological heterogeneity (3, 4). Following an initial HF hospitalization, death and recurrent events remain common even with guideline-directed therapy (5). At advanced stages, the short-term prognosis may be poor enough that accurate estimation of life expectancy becomes central to decisions regarding device implantation, transplantation referral, and palliative care (6).

Biomarkers are valuable because they convert diffuse clinical impressions into measurable signals. Natriuretic peptides and cardiac troponins remain essential for risk assessment. However, each marker captures only a portion of mortality risk, particularly in older patients (7). HF-related death may result from progressive pump failure, sudden arrhythmia, or cardiorenal collapse (8). A single pressure-volume or necrosis marker cannot encompass this complexity (9). Advances in prognostic methods and testing technologies have shifted biomarker research from single-marker prediction toward mechanism-informed risk stratification. Plasma proteomic clustering can identify HF subgroups with distinct outcomes that clinical clustering alone cannot detect (10). Metabolomic biomarkers significantly improve long-term mortality risk calibration in older patient cohorts (11). Furthermore, multi-omics integration provides a framework linking circulating molecules to pathophysiology and precision medicine (9).

Older patients with HF often show marked discordance between cardiac phenotype and clinical outcomes (12). For this population, accurate survival prediction informed by pathophysiological mechanisms is critical to guide therapy intensity and palliative care referral discussions (13). Although prior reviews have summarized individual HF biomarkers, few have systematically organized prognostic markers around aging-related pathophysiological pathways or focused specifically on their utility in older, multimorbid populations (3, 4). This narrative review synthesizes evidence on novel blood-based biomarkers associated with HF mortality and maps each circulating molecule to specific pathways of cardiac aging and systemic disease. On this basis, we propose a mechanistic conceptual framework for hypothesis generation and prospective validation in older adults with HF.

### 1.1 Literature search strategy

An initial targeted search of PubMed was conducted in December 2024 and covered articles published from database inception to the search date. An updated search was performed on 22 June 2026 using the same strategy to identify articles published between 1 January 2025 and 22 June 2026. The search combined terms related to heart failure, biomarkers, proteomics or metabolomics, and prognostic outcomes, including mortality, survival, and prognosis. Studies evaluating biomarkers in relation to survival or other prognostic outcomes in patients with heart failure were considered eligible, with particular attention given to studies involving older adults or addressing relevant mechanistic pathways. Non-English publications, studies restricted to *in vitro* cellular experiments, and preliminary exploratory studies that did not assess prognostic outcomes were excluded. The most relevant eligible studies were included in the narrative synthesis.

## 2 Circulating biomarkers and mechanistic signals in the aging heart

Aging fundamentally alters the clinical interpretation of HF biomarkers. Older patients commonly exhibit chronic low-grade inflammation, increased vascular stiffness, sarcopenia, impaired renal clearance, and endocrine remodeling (14). These factors perturb baseline biomarker concentrations and confer new physiological significance upon acute fluctuations. A mechanism-driven approach does not dismiss this biological complexity as noise; instead, it uses biomarkers to identify which pathological pathways dominate an individual patient's mortality risk. Throughout this review, evidence is classified into three categories (Table 1). It is described as older-adult-specific when recruitment was age-restricted or age effects were prespecified, as older-adult-enriched when the cohort had an advanced mean or median age without a dedicated age comparison, and as extrapolated when it was derived from broader HF populations.

**Table 1:** Integrated evidence map of pathway-based blood biomarkers for mortality risk in heart failure.

Pathway	Biomarkers	Prognostic evidence	Mechanistic interpretation	Potential interpretive role	Aging evidence
Fibro-Inflammatory Remodeling & Stress	sST2, galectin-3, GDF-15, H-FABP	Predicts HF severity, adverse outcomes, and mortality	Indicates myocardial strain, macrophage-driven fibrosis, and active cardiomyocyte injury	Defines a remodeling/stress axis; identifies high-risk structural convergence profiles	OE: GDF-15; EX: sST2, galectin-3, H-FABP.
Congestion, Endothelial & Vascular Aging	Endothelin-1, Bio-ADM, MR-proADM, CA125, adhesion molecules	Predicts decompensation, RV dysfunction, and mortality	Reflects vascular stiffness, endothelial leak, serosal inflammation, and right-sided pressure overload	Detects fluid redistribution and vascular stress before overt clinical edema develops	OS: CA125; OE: CA125 profiles, CA125/CRP; EX: Endothelin-1, bio-ADM, MR-proADM, adhesion molecules.
Cardiorenal & Multiorgan Reserve	NGAL, Cr/Alb ratio, albumin, PNI, De Ritis ratio, FT3/FT4 ratio	Predicts outcomes and mortality across HF populations	Captures macro-organ functional decline, cardiorenal continuum, and systemic frailty	Highlights mortality driven by systemic multiorgan failure rather than isolated cardiac issues	OS: De Ritis ratio; OE: NGAL, albumin, FT3/FT4 ratio; EX: Cr/Alb ratio, PNI.
Metabolic Stress & Inflammation	FGF21, phenylalanine, kynurenine, IL-6, hsCRP, NLR, IL-8, HMGB1	Predicts short/long-term mortality and advanced disease progression	Shows micro-level mitochondrial dysfunction, dysregulated amino acid catabolism, and chronic inflammaging	Identifies systemic metabolic exhaustion, cellular tissue damage, and cachexia trajectories	EX: FGF21, phenylalanine, kynurenine, IL-6, hsCRP, NLR, IL-8, HMGB1.
Proteomic & Metabolomic Phenotyping	Proteomic clusters, metabolomic panels	Outperforms standard clinical clustering; improves risk calibration	Identifies precise, underlying molecular phenotypes	Serves as discovery tools; requires translation into interpretable bedside panels	OS: metabolomic panels (mortality model); OE: metabolomic panels (end-stage profiling); EX: proteomic clusters and other metabolomic panels.

sST2, soluble suppression of tumorigenicity 2; GDF-15, growth differentiation factor 15; H-FABP, heart-type fatty acid-binding protein; Bio-ADM, bioactive adrenomedullin; MR-proADM, mid-regional pro-adrenomedullin; CA125, carbohydrate antigen 125; NGAL, neutrophil gelatinase-associated lipocalin; Cr/Alb, creatinine-to-albumin ratio; PNI, prognostic nutritional index; FT3/FT4, free triiodothyronine-to-free thyroxine ratio; FGF21, fibroblast growth factor 21; IL-6, interleukin-6; hsCRP, high-sensitivity C-reactive protein; NLR, neutrophil-to-lymphocyte ratio; IL-8, interleukin-8; HMGB1, high-mobility group box 1; HF, heart failure; RV, right ventricular; OE, older-adult-enriched evidence; EX, evidence extrapolated from broader HF populations; OS, older-adult-specific evidence; CRP, C-reactive protein.

Available evidence provides a rationale for considering this pathway-based paradigm. Fibrosis markers (e.g., galectin-3) identify maladaptive matrix remodeling (15–17). Vascular peptides [e.g., mid-regional pro-adrenomedullin (MR-proADM)] reflect endothelial hyperpermeability and systemic congestion (18–20). Renal and hepatic markers capture declining organ reserve (21–23). Immune and metabolic signals [e.g., growth differentiation factor 15 (GDF-15)] reveal systemic frailty and the senescence-associated secretory phenotype (24–29). Taken together, this evidence supports a transition in HF biomarker research away from identifying the single strongest univariate predictor of mortality. The emphasis is shifting toward characterizing which dysregulated pathways drive an individual patient's risk and whether these pathways can be reliably quantified in peripheral blood.

These pathway signals should be interpreted alongside clinical phenotypes. Acute decompensation is characterized by congestion, tubular stress, and inflammatory injury (30). Chronic heart failure with reduced ejection fraction (HFrEF) features systolic remodeling, neurohormonal activation, residual ischemia, and arrhythmic substrate (31, 32). Heart failure with preserved ejection fraction (HFpEF), the dominant phenotype in older adults, is characterized by microvascular inflammation, myocardial stiffness, and systemic metabolic derangement (33). Right-sided HF with pulmonary hypertension adds venous, hepatic, and serosal congestion (34, 35). Viewing HF mortality as the convergence of myocardial injury and systemic biological aging facilitates the integration of HFpEF, right-sided vulnerability, and multi-omics risk models into a multidimensional prognostic framework (10, 29, 36). Figure 1 summarizes the proposed mechanistic organization. Table 1 maps the biomarkers to prognostic, mechanistic, and potential interpretive domains and labels aging evidence as older-adult-specific, older-adult-enriched, or extrapolated using the definitions above.

**Figure 1:**
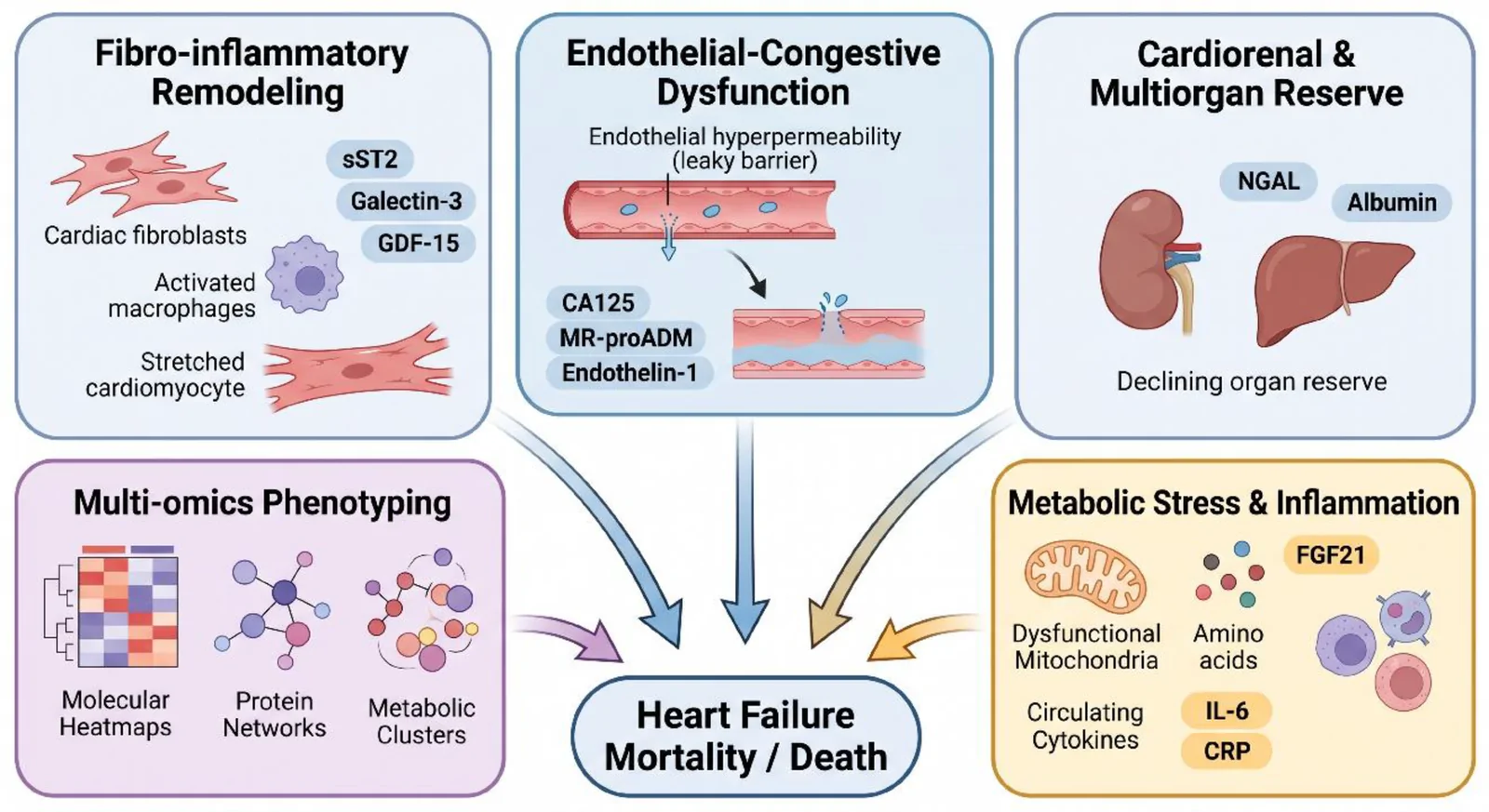
Mechanistic map linking blood biomarkers to mortality pathways in heart failure. The figure summarizes the review's framework in which heart failure mortality reflects the convergence of myocardial injury and systemic biological aging. Representative biomarkers are organized into five interconnected domains: (i) fibro-inflammatory remodeling and myocardial stress, represented by soluble suppression of tumorigenicity 2 (sST2), galectin-3, and growth differentiation factor 15 (GDF-15); (ii) endothelial-congestive dysfunction and vascular aging, represented by carbohydrate antigen 125 (CA125), mid-regional pro-adrenomedullin (MR-proADM), and endothelin-1; (iii) cardiorenal and multiorgan reserve, represented by neutrophil gelatinase-associated lipocalin (NGAL) and albumin; (iv) metabolic stress and inflammation, represented by fibroblast growth factor 21 (FGF21), interleukin-6 (IL-6), and C-reactive protein (CRP); and **(v)** proteomic and metabolomic phenotyping. Arrows indicate hypothesized biological convergence toward heart failure mortality and do not imply validated causal pathways or a fixed clinical biomarker panel.

### 2.1 Fibro-inflammatory remodeling and myocardial stress

Fibro-inflammatory remodeling represents one of the clearest links between molecular biology and HF mortality (37). Aging-associated chronic low-grade inflammation (inflammaging) and cellular senescence drive immune and fibroblast activation as well as maladaptive extracellular matrix remodeling, thereby accelerating myocardial fibrosis and functional decline (38).

Soluble suppression of tumorigenicity 2 (sST2) is released in response to mechanical stretch and inflammation (39). It acts as a soluble decoy receptor for interleukin-33, thereby attenuating the cardioprotective interleukin-33/transmembrane ST2 signaling axis (40). Prognostically, elevated sST2 repeatedly predicts disease severity and mortality in both acute and chronic HF cohorts (41–43). Mechanistically, it may reflect myocardial strain, inflammation, and extracellular matrix remodeling rather than a single disease-specific process (40). Its prognostic association appears relatively stable across age, body mass index, and renal-function strata (44), but this finding was derived mainly from broader HF cohorts and is therefore extrapolated to older adults. However, a preserved association across age strata does not establish older-adult-specific calibration, universal incremental value, or a treatment benefit of sST2-guided management.

Galectin-3 complements sST2 as a biomarker associated with macrophage activation and fibrotic remodeling. Studies have linked galectin-3 to fibroblast activation, collagen deposition, and adverse remodeling (15, 16). However, galectin-3 is not cardiac-specific: its concentration is influenced by renal function, age, systemic inflammation, and extracardiac fibrosis (45), which limits mechanistic interpretation in the cardiac context. Longitudinal data demonstrate that persistently elevated galectin-3 is associated with worse cardiovascular outcomes (17). Recent evidence supports galectin-3 for risk stratification in HF, yet variation in assays, endpoints, and HF phenotypes precludes a single, broadly applicable cutoff (46, 47).

GDF-15 is a broad cellular stress-responsive cytokine induced by inflammation, tissue injury, and metabolic stress (48). Beyond its role as a marker of these processes, GDF-15 exerts anorectic effects by binding the hindbrain receptor GFRAL and activating its co-receptor RET (24). This signaling reduces food intake and may contribute to weight loss and metabolic alterations. Prognostically, higher GDF-15 is associated with all-cause mortality in population studies (25) and with adverse outcomes in acute HF and HFrEF after adjustment for conventional covariates (26–28). HFpEF studies have reported associations in older and comorbid populations (29, 49). In chronic HFrEF populations, it may mark residual systemic risk beyond systolic remodeling, whereas in older patients with acute decompensated HFpEF it appears tightly linked to anemia, renal dysfunction, inflammation, and malnutrition (27, 29, 49).

The mechanistic significance of GDF-15 also explains its limitations. GDF-15 is not cardiac-specific and can be elevated in malignancy, renal disease, infection, and other systemic stress states (50). Rather than limiting its clinical utility, this broad responsiveness positions GDF-15 as a robust marker of global biological reserve and systemic vulnerability. In older patients with HF, a high or rising GDF-15 concentration may indicate progression from compensated cardiac disease toward a systemic stress state characterized by anorexia and catabolism.

Heart-type fatty acid-binding protein (H-FABP) captures a different aspect of myocardial stress: ongoing or recurrent myocardial injury. H-FABP is released rapidly from cardiomyocytes and has been studied as an early injury marker in HF and acute coronary syndromes (51, 52). In acute HF, elevated H-FABP predicts adverse outcomes and can identify patients with concurrent myocardial injury and renal vulnerability (53). Unlike sST2 or galectin-3, H-FABP does not primarily describe remodeling; instead, it indicates membrane injury, ischemia, or metabolic stress in cardiomyocytes (54). This etiologic distinction is critical for interpreting H-FABP. In acute myocardial infarction complicated by HF, H-FABP levels are driven mainly by infarct size and the extent of myocardial necrosis (52, 53). In chronic ischemic cardiomyopathy, elevated H-FABP indicates residual subclinical ischemia and ongoing low-grade myocyte injury (52). In non-ischemic dilated cardiomyopathy, increased H-FABP reflects heightened wall stress and intracellular metabolic dyshomeostasis rather than coronary occlusion (52, 55). H-FABP should therefore be interpreted as a myocardial injur*y*-axis marker within a multimarker framework rather than as a disease-specific biomarker. However, this prognostic evidence derives largely from broader acute and chronic HF cohorts and remains extrapolated when applied to older adults.

Collectively, sST2, galectin-3, GDF-15, and H-FABP can be organized within a conceptual fibro-inflammatory and myocardial stress axis. This axis is biologically relevant to aging because senescent immune cells, impaired matrix repair, mitochondrial dysfunction, and reduced regenerative capacity may amplify minor injuries into irreversible functional decline. A high-risk profile is therefore unlikely to be fully captured by an isolated elevation of sST2 or GDF-15; rather, it may reflect the convergence of remodeling, inflammation, systemic stress, and myocardial injury (37).

### 2.2 Congestion, endothelial dysfunction, and vascular aging

Congestion is a leading cause of HF hospitalization and an important predictor of mortality (56). Contemporary reviews distinguish intravascular from tissue congestion and systemic from pulmonary distribution. Hemodynamic congestion denotes increased cardiac filling pressures caused by volume expansion or blood redistribution (57). Tissue and serosal congestion refer to fluid accumulation in the interstitium and pleural or peritoneal cavities (58, 59). Right-sided systemic congestion reflects elevated venous pressure affecting the abdominal organs and peripheral tissues (57). Endothelial dysfunction involves impaired barrier integrity, increased permeability, and abnormal vasomotor regulation (58). These processes may overlap or dissociate, and no single circulating biomarker has been validated to quantify all components (58).

B-type natriuretic peptide (BNP) and N-terminal pro-B-type natriuretic peptide (NT-proBNP) primarily reflect myocardial wall stress induced by pressure or volume overload (60). Although established for diagnosis and prognosis, they do not directly quantify intravascular volume or filling pressure and are affected by age, renal dysfunction, and atrial fibrillation (59). Some patients with HFpEF have normal natriuretic peptide concentrations despite objective evidence of elevated filling pressures (61). In the GUIDE-IT trial, NT-proBNP-guided therapy did not reduce HF hospitalization or cardiovascular mortality compared with usual care in high-risk patients with HFrEF (62).

Carbohydrate antigen 125 (CA125) reflects mesothelial responses to mechanical and inflammatory stimuli but is not cardiac-specific and has a half-life of 5–12 days (58). Prognostically, CA125 has been associated with right ventricular dysfunction and adverse outcomes in HFpEF (36), with outcomes in stable chronic HF (63), and with mortality in acute HF among very old patients (64). In older patients with acute HFpEF, the prognostic impact of C-reactive protein (CRP) may be modulated by CA125 levels, reinforcing the hypothesis of a dynamic interaction between systemic inflammation and venous congestion (65). In a prospective multicenter study, CA125 was independently associated with peripheral edema, pleural effusion, and inferior vena cava dilation, while NT-proBNP was not independently associated with peripheral edema (66). In acute HF with severe tricuspid regurgitation, CA125 outperformed NT-proBNP for risk stratification (67). CA125 is therefore valuable because it captures a clinically important compartment that natriuretic peptides may miss: serosal and right-sided congestion (68). However, a CA125-guided strategy reduced the composite endpoint through fewer HF rehospitalizations but did not reduce mortality (69).

Bioactive adrenomedullin (bio-ADM) promotes vasodilation and endothelial barrier stability and is released during volume and endothelial stress (70). Higher concentrations have been associated with edema, orthopnea, and jugular venous distension (70). In a hemodynamic study, bio-ADM correlated more strongly with mean right atrial pressure than with pulmonary artery wedge pressure (71). Bio-ADM and MR-proADM are not interchangeable: bio-ADM assays quantify the active peptide, whereas MR-proADM assays quantify a stable fragment derived from the same precursor (18–20). MR-proADM is therefore an indirect signal associated with activation of the adrenomedullin system rather than a direct measurement of endothelial barrier function. Prognostically, MR-proADM has been associated with congestion and mortality in acute and chronic HF (19, 20). Building on broader HF evidence, recent research has examined MR-proADM in the age-associated phenotype of transthyretin cardiac amyloidosis. Higher levels were independently associated with mortality and HF events and improved prediction beyond established staging systems (72), consistent with broader HF meta-analytic evidence (73).

Endothelin-1 is linked to vasoconstriction, vascular inflammation, pulmonary vascular load, and altered renal sodium handling (74). Prognostically, elevated endothelin-1 has been associated with adverse outcomes in HFrEF and acute HF (75, 76). In an acute HF cohort, endothelin-1 was associated with peripheral congestion and lower urinary sodium, but not with pulmonary congestion severity (77). Age-related arterial stiffness and microvascular dysfunction provide mechanistic plausibility for amplified endothelin-1 signaling in older patients (78).

As discussed in Section 2.1, sST2 appears to reflect fibro-inflammatory remodeling more closely than any specific congestion compartment (41–44). Circulating adhesion molecules may provide complementary information on endothelial-immune activation. In the DAPA-HF trial, cellular adhesion molecules were associated with adverse outcomes in chronic HF, indicating that leukocyte-endothelial interaction may carry prognostic information beyond hemodynamic load (79).

No fixed multimarker congestion panel has been prospectively validated for routine treatment decisions (58, 59). The proposed pathway-based workflow is therefore conceptual and hypothesis-generating: CA125 may reflect serosal and right-sided congestion, bio-ADM may reflect venous congestion, MR-proADM may reflect adrenomedullin system activation, endothelin-1 may reflect vascular stress, and adhesion molecules may reflect vascular inflammation. These biomarkers should be interpreted alongside physical examination, natriuretic peptides, kidney function, and diuretic response (80). Prospective studies should determine whether prespecified panels provide incremental prognostic value and improve treatment decisions or clinical outcomes.

### 2.3 Cardiovascular-kidney-metabolic syndrome and multiorgan reserve

In older patients with HF, mortality risk is shaped not only by cardiac dysfunction but also by multimorbidity and frailty (13). This concept aligns with the emerging cardiovascular-kidney-metabolic syndrome framework, which emphasizes bidirectional links among cardiovascular disease, kidney dysfunction, metabolic abnormalities, and adverse clinical outcomes (81). In older patients with HF, renal, hepatic, endocrine, and nutritional biomarkers are therefore not secondary details. They may identify a high-risk phenotype in which mortality is driven by multiorgan vulnerability rather than ventricular dysfunction alone (7, 8).

Neutrophil gelatinase-associated lipocalin (NGAL) is a blood and urine marker linked to tubular injury, inflammation, and iron handling (21). Prognostically, elevated plasma NGAL was associated with mortality in an older chronic-HF cohort and with adverse outcomes in broader acute-HF cohorts (22, 23). NGAL may signal tubular stress before serum creatinine rises and is particularly useful when worsening renal function is clinically ambiguous, although its incremental utility over creatinine in acute HF remains inconsistent (82–84).

Creatinine-based measures remain important, but ratios and combined indices may reflect systemic reserve differently from isolated kidney function. The serum creatinine/albumin ratio has been associated with 28-day mortality in HF (85). In chronic kidney disease cohorts, models that integrate renal and clinical variables predict incident HF and all-cause mortality, underscoring the cardiorenal continuum (86). These findings suggest that integrated reserve metrics may complement single-organ labels.

Albumin and nutritional scores have emerged as highly informative prognostic tools in older patients with HF. The prognostic nutritional index has been associated with outcomes across HFrEF and HFpEF populations (87). In older patients with acute HF, the combination of clinical frailty-oriented scores with serum albumin improved one-year mortality prediction (88). Mechanistically, albumin may reflect nutrition, inflammation, hepatic synthesis, and capillary leakage (89, 90). Low albumin in HF may therefore reflect malnutrition, inflammatory burden, and vascular permeability simultaneously.

Hepatic and thyroid markers extend the reserve concept. Meta-analytic evidence links liver biomarkers to prognosis in HFpEF (91). The De Ritis ratio (aspartate aminotransferase/alanine aminotransferase ratio) has been associated with one-year major adverse cardiovascular events in older patients with acute decompensated HF (92). The free triiodothyronine (FT3)/free thyroxine (FT4) ratio has been proposed as a metabolic marker of frailty and prognosis in older adults with HF (93). Although these markers are not novel in laboratory medicine, their integration into HF mortality assessment is increasingly relevant.

Biological age may integrate these reserve domains. A recent study examined aerobic exercise training, biological age, and mortality together in chronic HFrEF (94). This study suggests that blood-derived aging measures may reveal whether a patient's biological age exceeds the chronological age and whether modifiable interventions can reduce that risk. This direction is particularly important because HF mortality is not only a cardiology outcome but also an outcome of aging biology. However, although aging-focused, the biological-age analysis used the broader HF-ACTION trial population (mean age 58.8 years) rather than an older age cohort (94).

### 2.4 Metabolic stress and inflammation

Metabolic biomarkers connect HF mortality to energy substrate utilization, mitochondrial stress, amino acid handling, and catabolism. Fibroblast growth factor 21 (FGF21) is a hepatokine and stress-responsive metabolic regulator that affects glucose and lipid metabolism (95). In broader HF cohorts, circulating FGF21 has been proposed to define a cardiohepatic stress circuit and has been associated with short-term prognosis (96, 97); subsequent studies have also linked elevated FGF21 to advanced disease and adverse outcomes (98). However, experimental studies suggest that FGF21 may exert cardioprotective effects (99). This discrepancy suggests that the elevation is compensatory: in human HF, FGF21 acts primarily as a distress signal of systemic metabolic stress and depleted reserve rather than as a marker of active cardioprotection (98, 99).

Circulating amino-acid and metabolite signatures may provide information on cardiac aging, disease severity, and prognosis. Dysregulated phenylalanine catabolism has been implicated in cardiac aging (100), while elevated phenylalanine was associated with mortality in critically ill patients with HF (101). Kynurenine has been associated with HF severity and complications (102). Beyond individual metabolites, targeted metabolomic profiling has linked circulating metabolite patterns to survival in HF (103). Taken together, these findings suggest that circulating metabolite profiles may improve metabolic risk stratification and provide prognostic information beyond conventional clinical variables (103).

Inflammation is not a single pathway; it includes acute-phase response, myeloid activation, cytokine signaling, endothelial adhesion, and adaptive immune remodeling (104, 105). A meta-analysis supported the prognostic value of systemic inflammatory biomarkers in HF, including interleukin-6 (IL-6), high-sensitivity CRP, and the neutrophil/lymphocyte ratio (106). High interleukin-8 (IL-8) serum levels independently predicted long-term all-cause mortality in chronic HF (107). Plasma high-mobility group box 1 (HMGB1), a key mediator of myocardial proinflammatory cytokine release, has been evaluated as a marker of clinical outcome in chronic HF (108).

These inflammatory markers differ in biological meaning: IL-6 and CRP indicate systemic inflammatory tone, IL-8 relates to neutrophil recruitment and chemotaxis, and HMGB1 suggests tissue damage and sterile inflammation (106–108). When combined with remodeling, congestion, or injury markers, they may aid phenotypic interpretation (109). This distinction is important because mortality mechanisms differ. For example, patients with high CA125 and MR-proADM may die from recurrent congestion and vascular decompensation, whereas those with high IL-6, low albumin, and altered amino acid metabolites may instead follow trajectories dominated by cachexia, infection vulnerability, or frailty-related decline (87, 88, 110, 111).

### 2.5 Proteomic and metabolomic phenotyping

Multi-omics approaches can identify candidate markers and molecular phenotypes that single markers may not capture. Proteomic clustering has been shown to outperform clinical clustering in identifying patients with HF who have distinct outcomes (10). Proteomic pathways across the ejection fraction spectrum in patients with HF and diabetes have provided insight into phenotype-specific biology (112). Proteomic biomarkers have also been linked to corrected QT interval in chronic HF, suggesting possible connections between molecular stress and arrhythmic substrate (113).

Metabolomics is advancing in parallel, but the aging evidence is heterogeneous. A UK Biobank analysis restricted to patients aged 60 years or older provides older-adult-specific evidence that metabolomic biomarkers improve 10-year mortality risk calibration and clinical utility (11). A prospective cohort study showed differential metabolomic prediction of outcomes in HFpEF and HFrEF (110). Older patients with end-stage HF also show distinct metabolomic profiles (111). Reviews support metabolomics as a research tool for HF risk stratification (114).

Multi-omics integration and machine learning are therefore best applied as discovery and stratification tools. A recent review supports multi-omics integration as a strategy for linking molecular mechanisms to precision medicine in HF (9). Machine learning approaches have also been proposed for risk stratification in HFpEF (115). The next step is to convert these prediction models into interpretable pathway-based panels that can be applied in routine HF care. Despite their promise, multi-omics approaches remain limited by assay heterogeneity, insufficient external and age-specific validation, uncertain incremental clinical utility, and limited bedside interpretability. Their use therefore remains largely exploratory, particularly in older HF populations.

## 3 Clinical translation: from biomarker lists to pathway-based panels

A clinically useful mortality biomarker strategy should begin by identifying the clinical decision to be addressed. For older patients hospitalized with acute HF, the key decisions may involve the intensity of decongestion, post-discharge monitoring, rehabilitation planning, or discussions about palliative care referral (13). For stable chronic HF, the decision may involve therapy optimization, amyloidosis evaluation, device timing, or follow-up interval (12). The biomarker panel should therefore match the clinical decision rather than maximize the number of measured molecules.

A practical blood-based panel should be tailored to the clinical question and prioritize complementary pathophysiological information; no fixed one-marker-per-pathway rule or universal cutoff is currently established (56, 109). Natriuretic peptides and troponins provide conventional hemodynamic and injury context. sST2 or galectin-3 may indicate remodeling. GDF-15 may reflect systemic stress. CA125 or MR-proADM may indicate congestion and endothelial dysfunction. NGAL or the creatinine/albumin ratio may reflect cardiorenal reserve. Albumin or the prognostic nutritional index may indicate nutrition and frailty. Selected inflammatory or metabolic markers may be added when the phenotype suggests cachexia (116). Figure 2 presents an illustrative, pathway-based panel.

**Figure 2:**
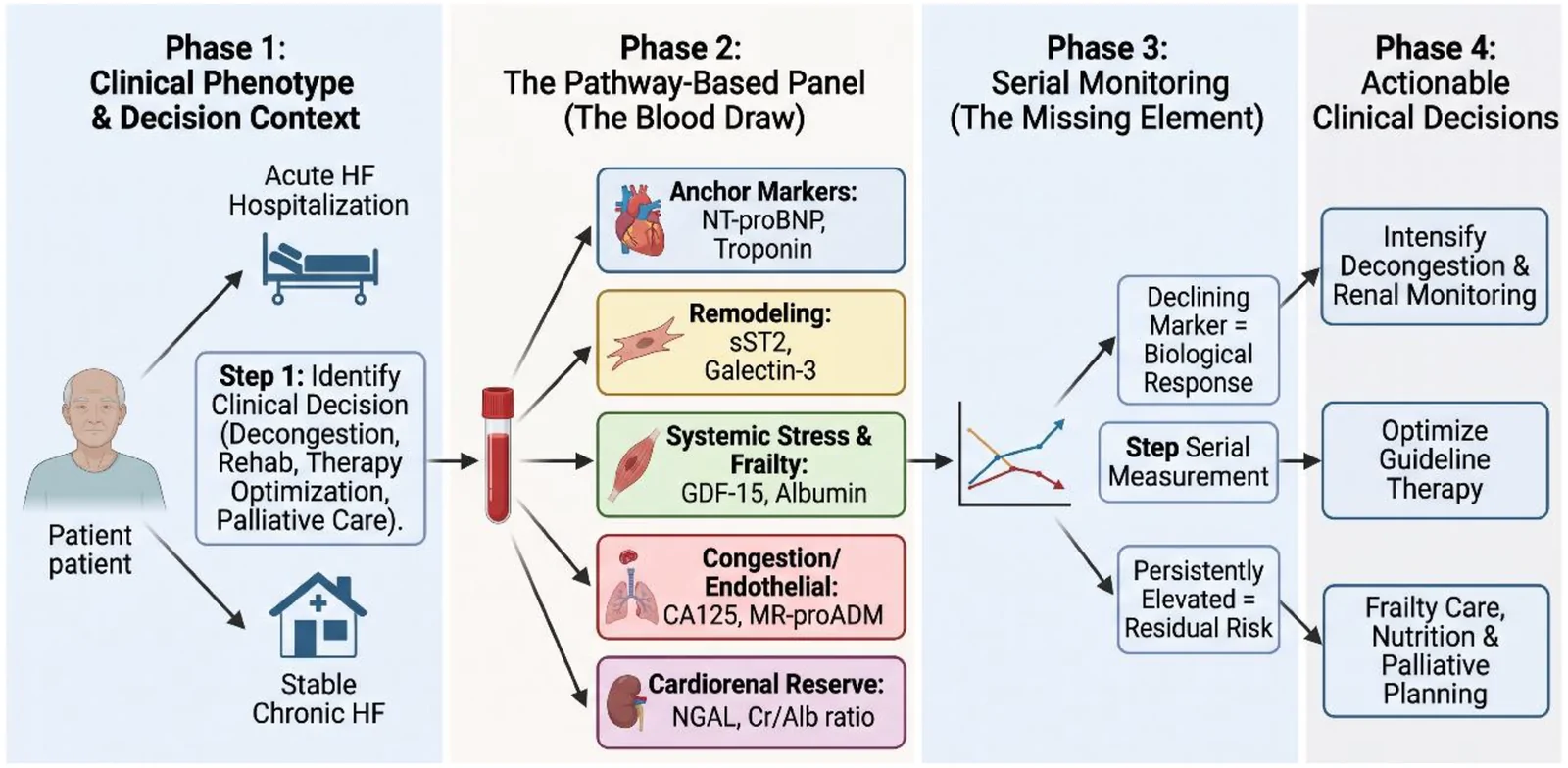
Conceptual workflow for pathway-based interpretation of blood biomarkers in heart failure mortality risk stratification. In Phase 1, the workflow begins with an assessment of the clinical scenario, phenotype, and decision context, covering two common clinical settings: acute heart failure hospitalization and stable chronic heart failure. In Phase 2, established biomarkers, including N-terminal pro-B-type natriuretic peptide (NT-proBNP) and troponin, provide the conventional hemodynamic and myocardial injury context, while candidate complementary biomarkers are organized according to the relevant pathophysiological pathways: remodeling, represented by soluble suppression of tumorigenicity 2 (sST2) and galectin-3; systemic stress and frailty, represented by growth differentiation factor 15 (GDF-15) and albumin; congestion and endothelial dysfunction, represented by carbohydrate antigen 125 (CA125) and mid-regional pro-adrenomedullin (MR-proADM); and cardiorenal reserve, represented by neutrophil gelatinase-associated lipocalin (NGAL) and the creatinine-to-albumin ratio (Cr/Alb). Phase 3 illustrates how serial measurements may distinguish declining biomarker concentrations, potentially indicating biological response, from persistently elevated concentrations, potentially indicating residual risk. Phase 4 illustrates how the integrated biomarker profile could inform future decisions concerning decongestion and renal monitoring, guideline-directed therapy, frailty and nutritional care, rehabilitation, and palliative planning. This workflow is illustrative and hypothesis-generating; no fixed biomarker panel, universal cutoff, or biomarker-guided treatment strategy has been prospectively validated. HF, heart failure.

Serial measurements may capture changes in congestion and hemodynamic status (117). A marker that remains elevated after apparent clinical stabilization may identify residual risk; conversely, a declining marker may indicate a biological response even before functional improvement becomes apparent. The challenge is to determine which changes are analytically reliable, biologically meaningful, and clinically actionable.

Implementation also requires caution. Many novel biomarkers predict mortality because they are correlated with age, renal dysfunction, inflammation, or disease severity. Incremental prognostic value must be tested against robust clinical models and standard biomarkers. Assays must be standardized, thresholds must be phenotype-specific, and models must be calibrated in older adults. A biomarker should not be adopted simply because it improves a statistical model; it should alter clinical action. Until older-adult-specific validation is available, associations derived from broader HF cohorts should not be used to infer geriatric cutoffs or treatment thresholds.

## 4 Evidence gaps and future directions

Several gaps limit current translation. First, many studies are observational and rely on single baseline measurements. Repeated-measurement designs are needed to determine whether biomarker trajectories predict death more accurately than static values. Second, HF phenotypes and etiologies are not interchangeable. A biomarker validated in HFrEF may not apply to HFpEF. Similarly, a marker associated with all-cause death may not predict sudden death, pump failure death, or infection-related death in the same manner. Third, aging biology should be treated as part of the HF phenotype. Future studies should incorporate frailty, cognitive status, nutritional reserve, and multimorbidity burden. A blood marker that appears nonspecific in a younger cohort may be highly informative in older patients with HF precisely because it captures systemic vulnerability. Fourth, biomarker-guided trials are needed. The field has many prognostic markers but limited data showing that measuring them improves outcomes. Finally, omics studies should prioritize interpretability and feasibility. Discovery panels can identify new proteins and metabolites, but clinical panels must be affordable, reproducible, and interpretable. The ideal future model is an interpretable blood-based pathway profile that tells clinicians whether the dominant risk is congestion, fibrosis, renal injury, inflammation, malnutrition, metabolic exhaustion, or a combination of these processes.

## 5 Conclusion

Novel blood-based biomarkers may provide complementary, non-interchangeable prognostic and mechanistic information across fibro-inflammatory remodeling, congestion, multiorgan reserve, metabolic stress, and molecular phenotyping. However, many candidates are influenced by cardiac and extracardiac conditions; their associations should not be equated with cardiac specificity, incremental prognostic value beyond established biomarkers, or treatment-guidance evidence. Evidence specific to older adults remains limited, and the pathway-based workflow proposed here is a conceptual, hypothesis-generating framework requiring age-specific calibration and prospective validation. An integrative approach may help characterize the convergence of cardiac injury and systemic biological aging, but its incremental value and ability to improve clinical decisions or outcomes require confirmation in biomarker-guided trials involving older, multimorbid HF populations.
